# Abrogation of lysophosphatidic acid receptor 1 ameliorates murine vasculitis

**DOI:** 10.1186/s13075-019-1973-0

**Published:** 2019-08-20

**Authors:** Chie Miyabe, Yoshishige Miyabe, Jun Nagai, Noriko N. Miura, Naohito Ohno, Jerold Chun, Ryoji Tsuboi, Hiroshi Ueda, Masayuki Miyasaka, Nobuyuki Miyasaka, Toshihiro Nanki

**Affiliations:** 1 Department of Rheumatology, Graduate School of Medical and Dental Sciences, Tokyo Medical and Dental University, Tokyo, Japan; 2 Department of Dermatology, Tokyo Medical University, Tokyo, Japan; 3 Division of Molecular and Pharmacology, Nagasaki University Graduate School of Biomedical Sciences, Nagasaki, Japan; 4 Laboratory for Immunopharmacology of Microbial Products, School of Pharmacy, Tokyo University of Pharmacy and Life Sciences, Tokyo, Japan; 5 Department of Molecular and Cellular Neuroscience, Dorris Neuroscience Center, The Scripps Research Institute, La Jolla, CA USA; 6 Interdisciplinary Program for Biomedical Sciences, Osaka University, Osaka, Japan; 7 Department of Clinical Research Medicine, Teikyo University, Tokyo, Japan; 8 Division of Rheumatology, Department of Internal Medicine, Toho University School of Medicine, 6-11-1 Omori-Nishi, Ota-ku, Tokyo, 143-8541 Japan

**Keywords:** Vasculitis, Lipid mediator, Neutrophil, Chemokine

## Abstract

**Background:**

Lysophosphatidic acid (LPA), generated by autotaxin (ATX), is a bioactive lipid mediator that binds to the receptors (LPA_1–6_), and serves as an important mediator in inflammation. Previous studies have demonstrated that LPA-LPA_1_ cascade contributes to arthritis and skin sclerosis. In this study, we examined the role of LPA signals in murine *Candida albicans* water-soluble fraction (CAWS)-induced vasculitis.

**Methods:**

ATX and LPA receptor expressions were analyzed by immunohistochemistry and quantitative reverse transcription-polymerase chain reaction. Effects of LPA_1_ inhibition on CAWS-induced vasculitis were evaluated in LPA_1_-deficient mice or using an LPA_1_ antagonist, LA-01. Migration activity was assessed using a chemotaxis chamber. The number of migrated fluorescently labeled neutrophils, which were transferred into the vasculitis mice, was counted in the aortic wall. CXCL1 and IL-8 concentrations were determined by enzyme-linked immunosorbent assay.

**Results:**

ATX and LPA_1_ were highly expressed in the inflamed region of CAWS-induced vasculitis. Severity of the vasculitis in LPA_1_-deficient mice was suppressed. The LPA_1_ antagonist, LA-01, also ameliorated the CAWS-induced vasculitis. LPA induced neutrophil migration, which was inhibited by LA-01 in vitro*.* Infiltration of transferred neutrophils from LPA_1_-deficient mice into the coronary arteries was suppressed. LA-01 also inhibited the infiltration of wild-type neutrophils. Expression of CXCL1 and IL-8 in human endothelial cells was enhanced by LPA, but was inhibited by LA-01. ATX and LPA_1_ expression levels were higher in the affected skin region of vasculitis patients than in healthy controls.

**Conclusions:**

These results suggest that LPA-LPA_1_ signaling contributes to the development of vasculitis via chemoattractant production from endothelial cells followed by neutrophil recruitment. Thus, LPA_1_ has potential as a novel target for vasculitis therapies.

## Introduction

Systemic vasculitides are characterized by the infiltration of inflammatory leukocytes into blood vessels, which induces destructive damage to the structures of the vessels. The affected vessels vary in size, type, and location according to the type of vasculitic disease [1]. For example, anti-neutrophil cytoplasmic antibody-associated vasculitis is well known to affect small vessels, Kawasaki disease middle vessels, and giant cell arteritis large vessels [1–3]. Immune cells, including neutrophils, macrophages, and lymphocytes, infiltrate the vessel wall and promote chronic inflammation in vasculitis [4]. Neutrophils have been shown to play an important role in vasculitis [5, 6]. They are activated in the inflamed vessel wall and release proteases, cytokines, chemokines, and neutrophil extracellular traps [5, 7]. Therefore, interfering with the recruitment and activation of neutrophils in the vessel walls is an underappreciated target for vasculitis therapies.

Autotaxin (ATX), which exhibits lysophospholipase D activity, generates lysophosphatidic acid (LPA) via the hydrolysis of lysophosphatidylcholine. LPA is a bioactive lipid mediator that binds to a group of cell surface G protein-coupled receptors (LPA_1–6_) [8–10]. We previously demonstrated that LPA_1_ plays an essential role in the pathogenesis of murine collagen-induced arthritis [11]. We also found that the LPA-LPA_1_ cascade contributes to the migration of macrophages into inflamed joints, Th17 differentiation, osteoclastogenesis, and the activation of fibroblast-like synoviocytes from rheumatoid arthritis [11, 12]. Previous studies reported that LPA_1_ was strongly expressed in the skin tissue of scleroderma patients, and the abrogation of LPA_1_ ameliorated murine bleomycin-induced skin sclerosis [13]. However, the role of the LPA-LPA_1_ cascade in the activation of neutrophils currently remains unclear. Moreover, the effects of LPA_1_ inhibition on vasculitis have yet to be examined.

Severe coronary arteritis accompanied by neutrophil infiltration was developed in the experimental model of murine *Candida albicans* water-soluble fraction (CAWS)-induced vasculitis, which is considered to be an appropriate model of arteritis [14–16]. We and others previously showed that neutrophils and macrophages play important roles in CAWS-induced vasculitis [17, 18].

In the present study, we demonstrate that LPA-LPA_1_ signals are essential for the development of vasculitis via neutrophil migration and also that the abrogation of LPA_1_ ameliorated CAWS-induced vasculitis, suggesting that LPA_1_ is a promising therapeutic target for vasculitis.

## Materials and methods

### Induction of CAWS-induced vasculitis

CAWS was prepared from *C. albicans* strain NRBC1385 using a previously described method [19]. Six-week-old male C57/BL6 mice were purchased from Oriental Yeast. The original lines of LPA_1_-deficient mice [20] were backcrossed to the inbred C57BL/6 strain for at least 15 generations. We then used these mice as the C57BL/6 background. In order to induce vasculitis, CAWS (1 mg/mouse) was injected intraperitoneally into mice in a volume of 0.2 ml once daily from days 1 to 5. On day 28, the heart was harvested and examined. The experimental protocol for animal experiments was approved by the Institutional Animal Care and Use Committee of Tokyo Medical and Dental University.

### Real-time reverse transcription-polymerase chain reaction (RT-PCR)

Total RNA was prepared from tissues including the aorta and coronary artery of CAWS-induced vasculitis and normal mice, and first-strand cDNA was synthesized. Quantitative real-time PCR was performed as described previously [21]. cDNA was amplified with primers for LPA_1–6_ and 18S ribosomal RNA (rRNA) as previously described [11]. 18S rRNA was used as an internal control in order to standardize the amount of sample mRNA, and the relative expression of real-time PCR products was determined.

### Immunohistochemistry

Paraffin-embedded skin tissues (4-μm-thick sections) from CAWS-induced vasculitis mice, vasculitis patients, and healthy donors were deparaffinized, immersed in 1 mM EDTA at 99–100 °C for 20 min, removed from the heat, and left to stand at room temperature for 20 min, followed by rinsing with a mixture of Tris-buffered saline with Tween 20. Endogenous peroxidase activity was blocked by an incubation in 0.3% H_2_O_2_ for 30 min. Sections were then blocked with 1% skim milk for 45 min and stained with a rabbit anti-ATX polyclonal antibody (pAb) (2 μg/ml; Cayman Chemical), -LPA_1_ pAb (10 μg/ml: Lifespan Biosciences), or normal rabbit IgG (Sigma Aldrich) as an isotype control at room temperature for 45 min. Antibody binding was detected using the Envision kit (DakoCytomation) as described previously [11].

### Treatment of CAWS-induced vasculitis with an LPA_1_ antagonist

Mice injected with CAWS were treated with an LPA_1_ antagonist (LA-01 [11, 12] provided by Ono Pharmacological; 200, 60 mg/kg/day, or vehicle) by oral gavage twice a day from days 0 to 28. On day 28, the fixed hearts were embedded in paraffin and sectioned. In order to observe histological changes in the coronary arteries and aorta in detail, step sections in a horizontal direction were prepared every 20 μm. Sections were stained with hematoxylin and eosin (H&E). In order to quantitatively evaluate vascular inflammation, each of the 5 areas (3 aortic root areas and both coronary arteries) was scored on a scale of 0–3 according to the classification system for the areas of cellular infiltration: (a) aortic root (score 0 for no inflammation; 1, cell infiltration < 100 μm in diameter; 2, 100–199 μm in diameter; 3, ≥ 200 μm in diameter) and (b) coronary arteries (score 0 for no inflammation; 1, cell infiltration < 50 μm in diameter; 2, 50–99 μm in diameter; 3, ≥ 100 μm in diameter). The severity of arteritis in each mouse was defined as the sum of the scores of the 5 segments (maximum possible score of 15).

### In vitro chemotaxis assay of neutrophils

The Ly-6G-positive neutrophils of wild-type (WT) or LPA_1_-deficient mice were purified from spleen using MACS microbead-coupled mAbs and magnetic cell separation columns (Miltenyi Biotec) as previously described [17]. The purity of Ly-6G-positive cells was more than 95%. The purified neutrophils from WT mice were incubated with LA-01 (0, 1, and 10 nM) at 37 °C for 30 min in RPMI 1640 medium (Sigma-Aldrich). After being incubated, neutrophils were washed twice with RPMI 1640. Neutrophils (1 × 10^6^ cells/well) were added to the upper well of the transwell with a 3.0-μm pore polycarbonate membrane insert (Corning), while LPA [1-Oleoyl LPA (Cayman Chemical)] (10 μM) was added to the lower wells, and then incubated at 37 °C for 2 h. The number of cells that migrated into the lower well was counted using the Accuri C6 Flow Cytometer (Accuri Cytometers). Triplicates of three independent experiments were performed.

### Migration of neutrophils into inflamed vascular walls

Murine Ly6G-positive splenocytes were labeled with Cell Tracker Green CMFDA (5-chloromethylfluorescein diacetate) (Molecular Probes), according to the protocol supplied by the manufacturer, and previously described [17]. The labeled cells (1.0 × 10^7^) were transferred into the tail vein of CAWS-induced vasculitis mice on day 21. Recipient mice were treated with LA-01 (100 mg/kg) or saline 12 h and 30 min before and 12 h after the transfer. Twenty-four hours after the transfer, the number of labeled cells in the synovium was counted under a fluorescent microscope (Biozero) as previously described [17].

### Enzyme-linked immunosorbent assay (ELISA)

Human umbilical vein endothelial cells (HUVECs) were cultured overnight in 48-well plates (1 × 10^5^ cells/well), then incubated with LA-01 (0, 0.2, 1, 10 nM) for 30 min before being stimulated with LPA (10 μM) in FCS-free endothelial growth basal medium-2 at 37 °C for 24 h. CXC chemokine ligand 1 (CXCL1) and IL-8 protein levels in the culture supernatant were assessed using ELISA kits (R&D Systems) according to the instructions supplied by the manufacturer.

### Patient specimens

The skin tissues of affected lesions were obtained from active vasculitis patients [microscopic polyangiitis (MPA), *n* = 5; polyangiitis nodosa (PN), *n* = 3; eosinophilic granulomatosis with polyangiitis (EGPA), *n* = 1] who fulfilled diagnostic criteria by the Research Group of Intractable Vasculitis, Ministry of Health, Labor, and Welfare of Japan [22] as well as from healthy controls [*n* = 3]. Tissues were stained with ATX and LPA_1_ as described above. The experimental protocol was approved by the Ethics Committee of the Tokyo Medical University, and all subjects provided informed consent according to the Declaration of Helsinki principles.

### Statistical analysis

Data are expressed as the mean ± SEM. A comparison of data from the two groups was conducted by the Student *t* test. *P* values less than 0.05 were considered to be significant.

## Results

### Expression of ATX and LPA receptors in murine CAWS-induced vasculitis

**Fig. 1:**
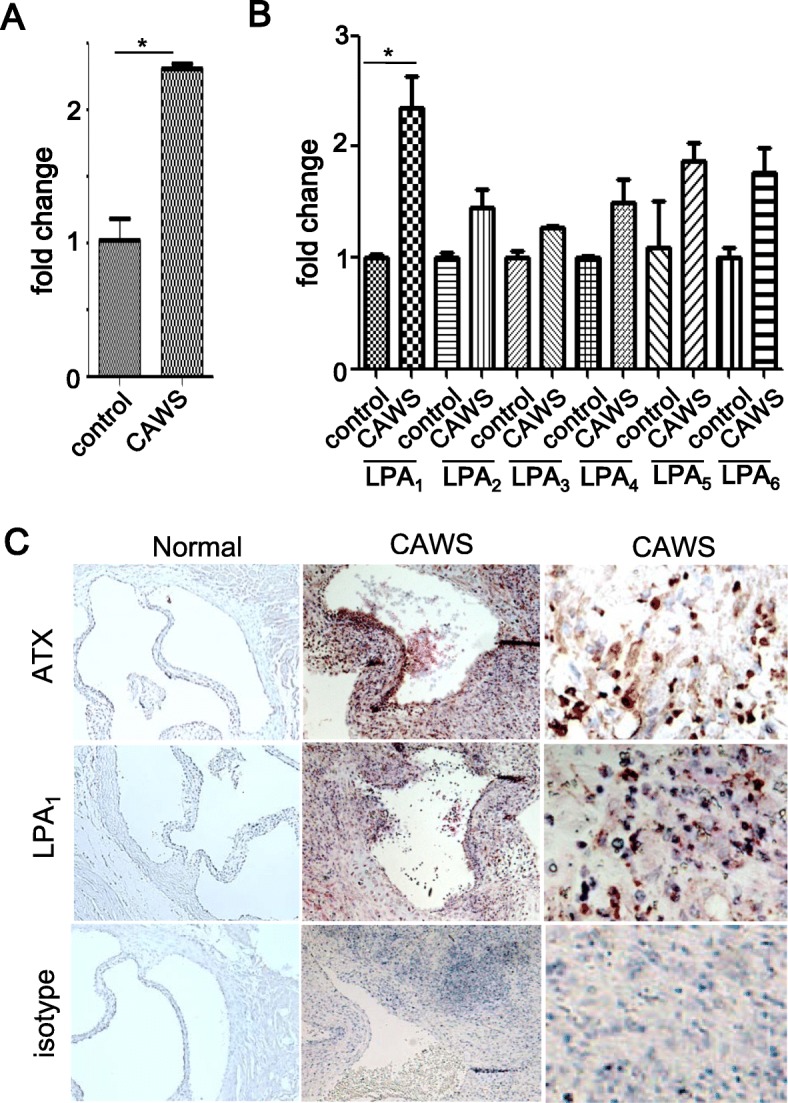
Expression of ATX and LPA receptors in CAWS-induced vasculitis. **a**, **b** Expression levels of ATX (**a**) and LPA_1–6_ (**b**) mRNAs were compared between CAWS-induced vasculitis mice (*n* = 3) and control mice (*n* = 3) using real-time RT-PCR. Data are the mean ± SEM; *P < 0.05 vs OA. Data are derived from multiple samples. **c** The expression of ATX and LPA_1_ in the aorta of control mice and CAWS-induced vasculitis mice was analyzed by immunohistochemistry. Original magnification: × 100 (left and middle panel), × 400 (right panel)

We analyzed the expression of ATX and the LPA receptors, LPA_1–6_, in murine CAWS-induced vasculitis using quantitative RT-PCR and immunohistochemistry. CAWS-injected mice showed inflammatory cell infiltration in the aortic root and coronary arteries [17]. The expression of ATX mRNA in the aorta and coronary arteries was significantly higher in CAWS-induced vasculitis mice than in control mice (Fig. 1a). The expression of each LPA receptor mRNA was compared between CAWS-induced vasculitis and control mice. The expression of LPA_1_ mRNA was increased in CAWS-induced vasculitis mice than in control mice (Fig. 1b). No significant differences were observed in the expression of LPA_2–6_ between CAWS-induced vasculitis and control mice. Immunohistochemical analysis revealed that ATX and LPA_1_ were highly expressed in infiltrated inflammatory cells in the aortic root and coronary arteries in CAWS-induced vasculitis mice than in control mice (Fig. 1c). These results suggest that the ATX-LPA-LPA_1_ cascade contributes to the pathogenesis of CAWS-induced vasculitis.

### Inhibition of inflammatory cell infiltration into the vascular wall by the abrogation of LPA_1_

**Fig. 2:**
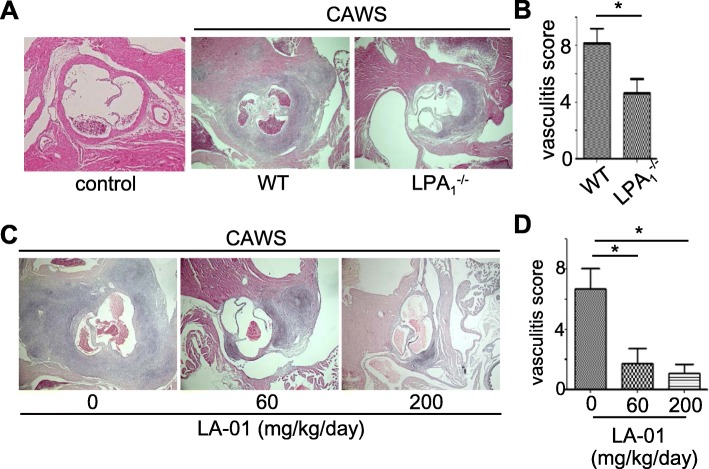
Inhibition of inflammatory cell infiltration into the vascular wall by the abrogation of LPA_1_. **a**, **b** C57/BL6 WT and LPA_1_-deficient mice were injected intraperitoneally with CAWS. The hearts of WT and LPA_1_-deficient mice (*n* = 10, each) were stained with H&E (**a**). Representative photomicrographs are shown. The vasculitis scores of WT and LPA_1_-deficient mice were evaluated (**b**). **c**, **d** C57/BL6 mice were injected intraperitoneally with CAWS from days 0 to 5. LA-01 (0, 60, 200 mg/kg/day; *n* = 10, each) was administered twice daily from days 0 to 28. On day 28, the hearts of mice were stained with H&E (**c**). Representative photomicrographs are shown. The vasculitis score was evaluated (**d**). Original magnification: × 40 (**a**, **c**). Data are the mean ± SEM. *P < 0.05 vs WT mice or vehicle (**b**, **d**)

We next examined the role of LPA_1_ in the development of vasculitis using LPA_1_-deficient mice (Fig. 2a, b)*.* LPA_1_-deficient mice were resistant to CAWS-induced vasculitis. Histologically, inflammatory cell infiltration was suppressed in the coronary arteries and aortic roots of LPA_1_-deficient mice, whereas abundant cellular infiltration was observed in WT mice.

In order to examine the effects of the LPA_1_ inhibitor on vasculitis, we treated CAWS-induced vasculitis mice with LA-01 (LPA_1_ antagonist) (Fig. 2c, d). LA-01 reduced the vasculitis score of mice treated with CAWS in a dose-dependent manner. These results suggest that LPA_1_ plays important roles in the development of vasculitis and can be considered as a potential new therapeutic target.

### LPA_1_ is essential for LPA-induced neutrophil migration

**Fig. 3:**
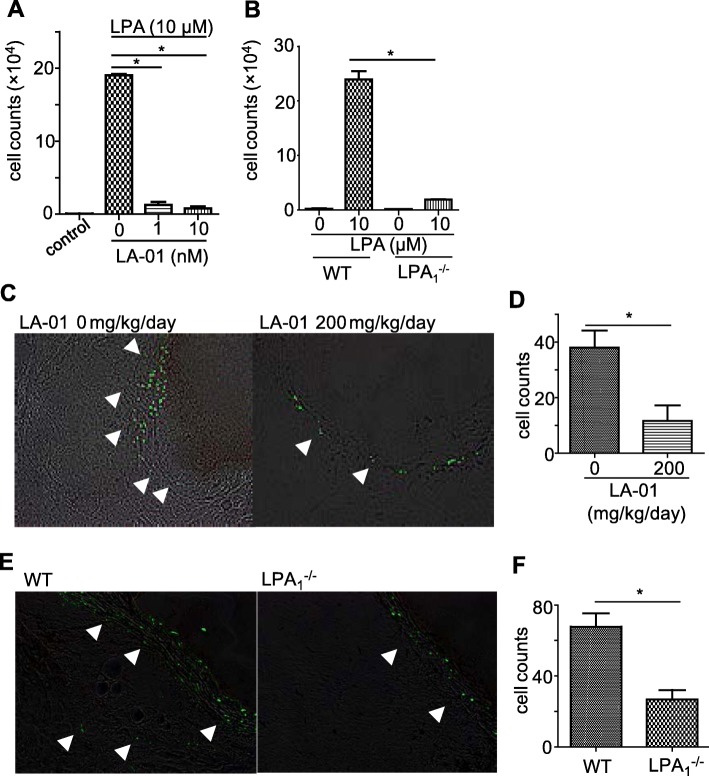
LPA_1_ is essential for LPA-induced neutrophil migration. **a** Ly-6G-positive neutrophils from murine spleen were incubated with LA-01 (0, 1, 10 nM) at 37 °C for 30 min in RPMI 1640. Cells were added to the upper wells of the transwell, LPA (10 μM) was loaded into the lower wells, and cells were incubated at 37 °C for 2 h. **b** Ly-6G-positive neutrophils from the spleen of WT or LPA_1_-deficient mice were loaded into the upper wells, LPA (10 μM) was added to the lower wells of the transwell, and cells were incubated at 37 °C for 2 h. The number of neutrophils that migrated into the lower well was counted by flow cytometry. Triplicates of three independent experiments were performed. Data are mean ± SEM; *P < 0.05 vs vehicle or WT mice. **c**, **d** Fluorescence-labeled Ly-6G-positive neutrophils from the spleen of WT mice were transferred into CAWS-induced vasculitis WT mice on day 21. The recipient mice were treated with LA-01 (100 mg/kg) or saline as a control 12 h and 30 min before and 12 h after the transfer. Migrated labeled cells were evaluated histologically 24 h after the transfer (**c**), and the number of labeled cells that migrated into the vascular walls was counted (**d**). **e**, **f** Fluorescence-labeled Ly-6G-positive neutrophils from the spleen of LPA_1_-deficient or WT mice were transferred into WT vasculitis mice on day 21. Migrated labeled cells were evaluated histologically 24 h after the transfer (**e**), and the number of labeled cells that migrated into the vascular walls was counted under a fluorescent microscope (**f**). Arrows indicate migrated labeled cells. Original magnification × 200 (**c**, **e**). Data are the mean ± SEM; *P < 0.05 vs vehicle or WT mice (**d**, **f**)

We next investigated the mechanism by which LPA drives vascular inflammation. We have previously shown that abundant neutrophils are infiltrating in the vascular wall in CAWS-induced vasculitis [17]. Therefore, we asked if LPA_1_ was important for LPA-induced neutrophil recruitment in vitro*.* Neutrophils were isolated from mouse spleen and stimulated with LPA (10 μM), which induced the migration of neutrophils in transwell chambers. This effect was remarkably suppressed by the treatment of LA-01 (Fig. 3a). In addition, the effect on neutrophil migration of LPA is abrogated in neutrophils derived from LPA_1_-deficient mice (Fig. 3b).

In order to determine the role of LPA_1_ on neutrophil recruitment in vivo, fluorescently labeled Ly-6G-positive neutrophils from WT mice were transferred intravenously into CAWS-induced vasculitis mice, and the number of labeled neutrophils that infiltrated the vascular walls was counted 24 h later. LA-01 (100 mg/kg) was administered 12 h and 30 min before and 12 h after the transfer. Although this short-term treatment of LA-01 did not ameliorate the vasculitis score, it suppressed de novo neutrophil migration into the inflamed vessels (Fig. 3c, d).

We also compared neutrophil recruitment into the vasculitis region between neutrophils derived from LPA_1_-deficient and WT mice. The number of migrated neutrophils derived from LPA_1_-deficient mice was lower than that from WT mice (Fig. 3e, f). These results indicate that the migration of neutrophils into inflamed vascular walls is dependent on LPA_1_.

### Effects of LPA-LPA_1_ signals on chemokine production from vascular endothelial cells

**Fig. 4:**
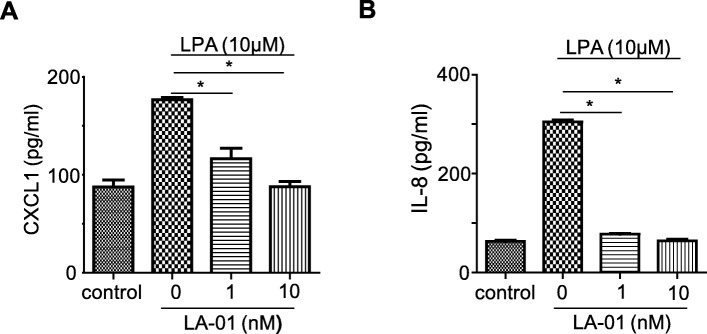
Effects of LPA-LPA_1_ signals on chemokine production from vascular endothelial cells. **a**, **b** HUVECs were stimulated with LPA (10 μM) at 37 °C for 24 h after being incubated with LA-01 (0, 0.2, 1, 10 nM) for 30 min. The protein levels of CXCL1 (**a**) and IL-8 (**b**) in the culture supernatant were assessed by ELISA. Data are the mean ± SEM; *P < 0.05 vs LA-01 (0 nM)

During inflammatory processes, chemokines upregulated on the surface of activated endothelial cells are thought to promote neutrophil recruitment [23]. We next examined the effects of LPA-LPA_1_ signals on chemokine production from endothelial cells. A stimulation with LPA induced the production of IL-8 and CXCL1 by HUVECs, which are the key chemoattractants of neutrophils [24]. The upregulated production of IL-8 and CXCL1 from LPA-stimulated HUVECs was inhibited by LA-01 (Fig. 4a, b).

### Expression of ATX and LPA_1_ in the skin region of vasculitis patients

**Fig. 5:**
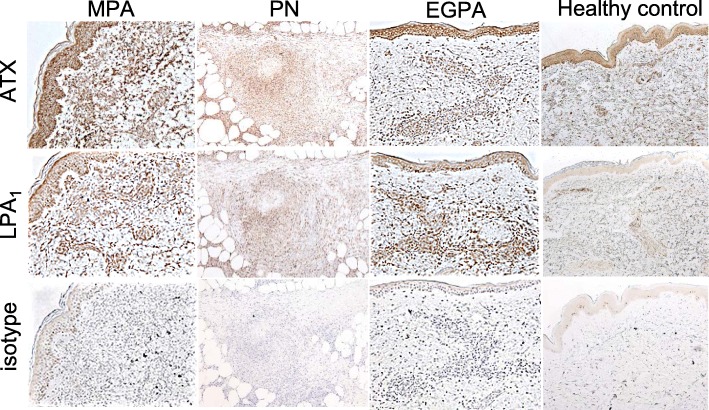
Expression of ATX and LPA_1_ in the skin region of vasculitis patients. The expression of ATX and LPA_1_ in the skin regions of vasculitis patients (MPA: *n* = 2, PN: *n* = 2, EGPA: *n* = 1) and healthy controls (*n* = 3) was analyzed by immunohistochemistry. Original magnification × 40

To assess the clinical relevance of our observations, we further analyzed the expression of ATX and LPA_1_ in the skin region of vasculitis patients by immunohistochemical analysis. Although no inflammatory changes were detected in skin tissue from healthy donors, ATX was expressed in the epidermis and in scattered cells in the dermis. The expression of LPA_1_ in normal skin was minimal. In contrast, ATX and LPA_1_ were strongly expressed in inflammatory cells, which were located in the dermis of skin tissues derived from MPA, EGPA, and PN patients (Fig. 5).

## Discussion

In the present study, we have demonstrated that ATX and LPA_1_ were strongly expressed in the heart tissues of CAWS-induced vasculitis mice as well as in skin samples from patients with vasculitis. In addition, inhibition of LPA_1_ ameliorated CAWS-induced vasculitis. LPA-LPA_1_ signals induced neutrophil migration in vitro and in vivo. LPA-LPA_1_ signals also upregulated chemoattractant production from endothelial cells. These results suggest that the LPA-LPA_1_ cascade plays important roles in the vascular inflammation via neutrophil recruitment.

We previously reported that neutrophils were necessary for the pathogenesis of CAWS-induced vasculitis [17]. In the present study, we demonstrated that LPA-LPA_1_ signals induced neutrophil migration in vitro as well as neutrophil recruitment into the inflamed vasculitis region in vivo. LPA also induced the production of CXCL1 and IL-8 in endothelial cells via LPA_1_. Thus, decreasing the production of CXCL1 and IL-8 in the inflamed vascular walls of CAWS-induced vasculitis by inhibiting LPA_1_ may also contribute to the suppression of neutrophil infiltration in vivo. However, the stimulation with LPA did not alter the production of reactive oxygen species or elastase by neutrophils in vitro (data not shown). Therefore, LPA-LPA_1_ signals may be direct and/or indirect inducers of neutrophil migration, but not neutrophil activation during vasculitis.

We reported that LPA_1_ contributed to Th17 cell differentiation and macrophage migration in type II collagen-induced arthritis [11]. T cells and macrophages have also been shown to accumulate in the vasculitis region of CAWS-induced vasculitis [17]. It has been shown that CAWS-injected mice had higher proportion of Th17 cells in the spleen, suggesting that an imbalance between regulatory T cell and Th17 may consequently lead to CAWS-induced vasculitis [18]. The LPA-LPA_1_ cascade may also contribute to the pathogenesis of vasculitis via Th17 cell differentiation and macrophage accumulation as well as neutrophil migration.

In the present study, we identified important roles for LPA_1_ in the inflammatory processes in CAWS-induced vasculitis. Although LPA_2–6_ were also expressed in the vasculitis region of CAWS-induced vasculitis mice, their roles in vasculitis currently remain unclear. Further studies are needed in order to determine the roles of LPA_2–6_ in the pathogenesis of vasculitis. ATX and LPA_1_ were also expressed in the affected regions of MPA, PN, and EGPA, as well as CAWS-induced vasculitis. The ATX-LPA-LPA_1_ cascade may also contribute to the pathogenesis of vasculitis in patients with MPA, PN, and EGPA. Our study clearly demonstrated that LPA-LPA_1_ pathway is important for the development of vasculitis in mouse and human, providing a rationale for the trial of new therapeutic strategy for vasculitis.

## Conclusions

In conclusion, the results of the present study suggest that LPA promotes neutrophil migration into the inflamed vascular walls of CAWS-induced vasculitis via LPA_1_. We consider LPA_1_ targeting therapy, supported further by other drug discovery efforts targeting LPA receptors [25] to have potential for the treatment of patients with vasculitis.

## Data Availability

The datasets used and/or analyzed during the current study are available from the corresponding author on reasonable request.

## Abbreviations

ATX: Autotaxin
CAWS: *Candida albicans* water-soluble fraction
CXCL1: CXC chemokine ligand 1
EGPA: Eosinophilic granulomatosis with polyangiitis
H&E: Hematoxylin and eosin
HUVECs: Human umbilical vein endothelial cells
LPA: Lysophosphatidic acid
MPA: Microscopic polyangiitis
pAb: Polyclonal antibody
PN: Polyarthritis nodosa
rRNA: Ribosomal RNA
RT-PCR: Reverse transcription-polymerase chain reaction
WT: Wild type
